# Sagittal slope angle of lateral atlantoaxial articulation is associated with the severity of basilar invagination with atlantoaxial dislocation and predicts reduction degree after surgery

**DOI:** 10.1186/s12891-024-07696-4

**Published:** 2024-07-24

**Authors:** Xia-Qing Sheng, Zi-Han Peng, Chen Ding, Bei-Yu Wang, Ying Hong, Peng-Fan Chen, Yang Meng, Hao Liu

**Affiliations:** 1 Department of Orthopedic Surgery and Orthopedic Research Institute, West China Hospital, Sichuan University, No. 37 Guo Xue Xiang, Chengdu, 610041 Sichuan China; 2 West China School of Nursing, Sichuan University/Department of Anesthesia and Operation Center, West China Hospital, No. 37 Guo Xue Xiang, Chengdu, 610041 Sichuan China; 3 West China School of Medicine, West China Hospital, Sichuan University, No. 37 Guo Xue Xiang, Chengdu, 610041 Sichuan China; 4 Department of Orthopedic Surgery, West China Hospital, Sichuan University, No. 37 Guo Xue Xiang, Chengdu, 610041 Sichuan China

**Keywords:** Atlantoaxial dislocation, Basilar invagination, Lateral atlantoaxial articulation, Predictors, Reduction degree, Sagittal slope angle

## Abstract

**Objective:**

To investigate (1) lateral atlantoaxial articulation (LAA) morphology in patients with basilar invagination (BI) with atlantoaxial dislocation (AAD) and healthy individuals and its relationship with the severity of dislocation and (2) the effect of the LAA morphology on reduction degree (RD) after surgery.

**Methods:**

In this retrospective propensity score matching case-control study, imaging and baseline data of 62 patients with BI and AAD from 2011 to 2022 were collected. Six hundred thirteen  participants without occipitocervical junctional deformity served as controls. Logistic regression and receiver operating characteristic (ROC) curve were used for analysis.

**Results:**

The age, BMI and sex did not differ significantly between the two groups after propensity score matching. Sagittal slope angle (SSA) and coronal slope angle (CSA) was lower and greater, respectively, in the patient group than in the control group. A negative SSA value usually indicates anteverted LAA. Regression analysis revealed a significant negative correlation between SSA and severity of dislocation. However, no relationship was found between CSA and the severity of dislocation. The multivariate logistic regression analysis revealed that minimum-SSA emerged as an independent predictor of satisfactory reduction (RD ≥ 90%). The ROC curve demonstrated an area under the curve of 0.844, with a cut-off value set at -40.2.

**Conclusion:**

SSA in patients group was significantly smaller and more asymmetric than that in the control group. Dislocation severity was related to SSA but not to CSA. Minimum-SSA can be used as a predictor of horizontal RD after surgery.

**Supplementary Information:**

The online version contains supplementary material available at 10.1186/s12891-024-07696-4.

## Introduction

Basilar invagination (BI) is an occipital cervical malformation caused by developmental abnormalities, often accompanied by atlantoaxial dislocation (AAD), atlantooccipital fusion, Chiari malformation, and Klippel-Feil syndrome [1–3]. In BI with AAD, pathology is characterized by a combination of “vertical dislocation” and “transverse dislocation,” in which the foramen dentata protrudes upward and backward into the foramen magnum, compressing the medulla oblongata and spinal cord, causing related symptoms [4]. Lateral atlantoaxial articulation (LAA) plays an important role in neck movements. Some researchers [5, 6] have observed sagittal and coronal slopes in LAA in patients with BI and AAD. These may cause horizontal and vertical dislocations [7]; however, there is insufficient evidence to date.

Although more and more posterior surgical strategies have been reported, the unsatisfactory reduction rate (reduction degree < 80% or < 90%) is approximately 14.5 -37.1% [8–10]. Surgeons must have a clearer and more objective understanding of the LAA slope in BI with AAD and its differences from that in healthy individuals, which requires objective indicators that can be easily measured and effectively reflect degree of dislocation.

 Therefore, the purpose of this study was to (1) explore the difference between the LAA slope angle in patients with BI with AAD and healthy people and its relation with dislocation severity and (2) determine whether slope angle can be used as a predictor of reduction degree (RD) after surgery.

## Methods

### Patient data

The local ethics committee approved this study. From January 2010 to February 2022, patients with BI with AAD and a control group without deformity or other diseases of the upper cervical spine were included from the hospital medical records and imaging system.

Inclusion criteria consisted of (1) age: 18–75 years, (2) complete three-dimensional computed tomography (CT) reconstruction of the occipitocervical junction area of the cervical spine. Exclusion criteria included (1) history of skull base or cervical spine surgery before admission to our hospital, (2) dislocation caused by tumor, infection and trauma, (3) bone fusion between atlas and axis.

**Fig. 1:**
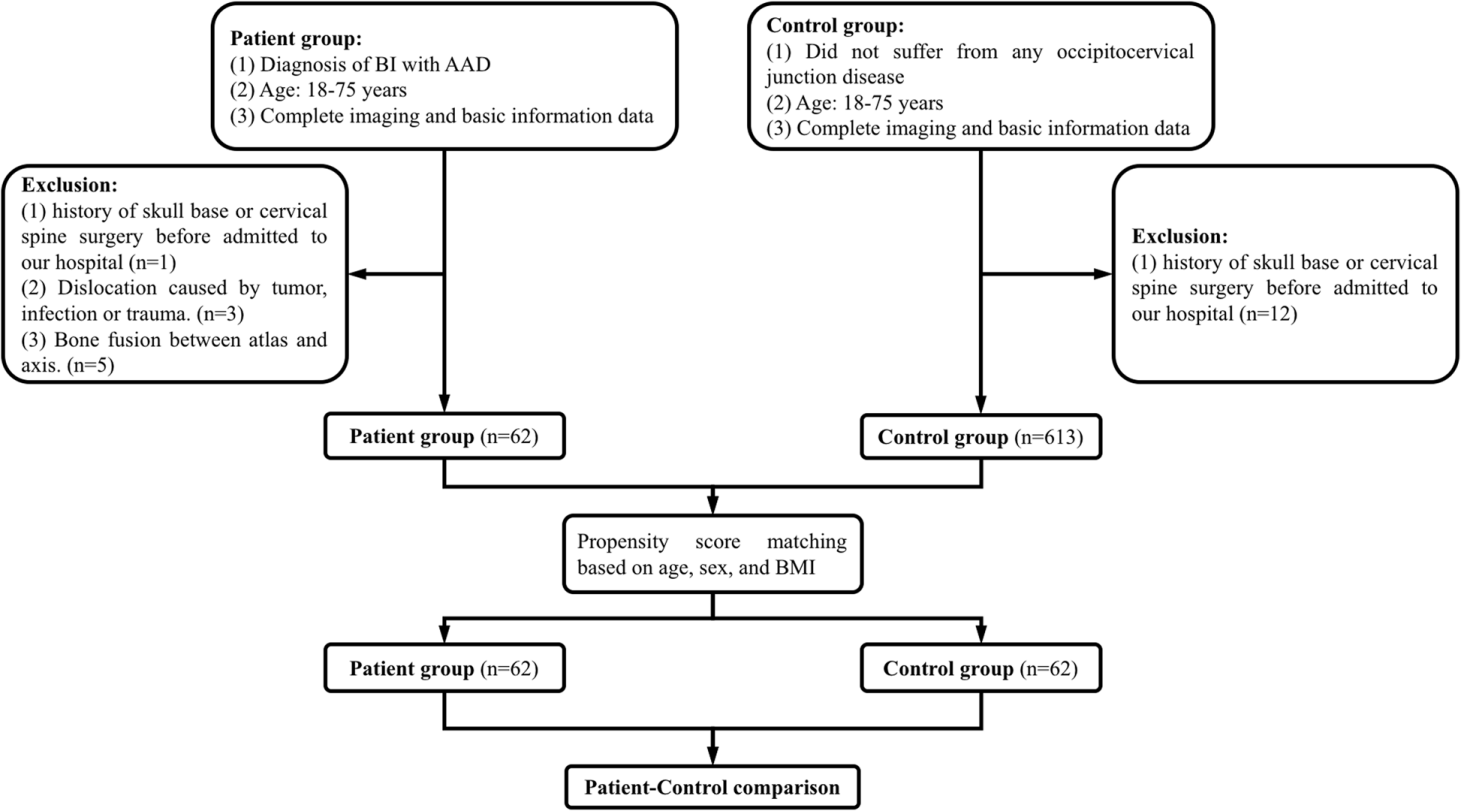
Flow diagram showing the process of patient selection and matching

Through propensity score matching method, those who did not suffer from occipitocervical junction disease and had completed three-dimensional CT reconstruction of the cervical spine and skull base were included from our hospital’s database as the control group (Fig. 1).

### Outcome measures

**Fig. 2:**
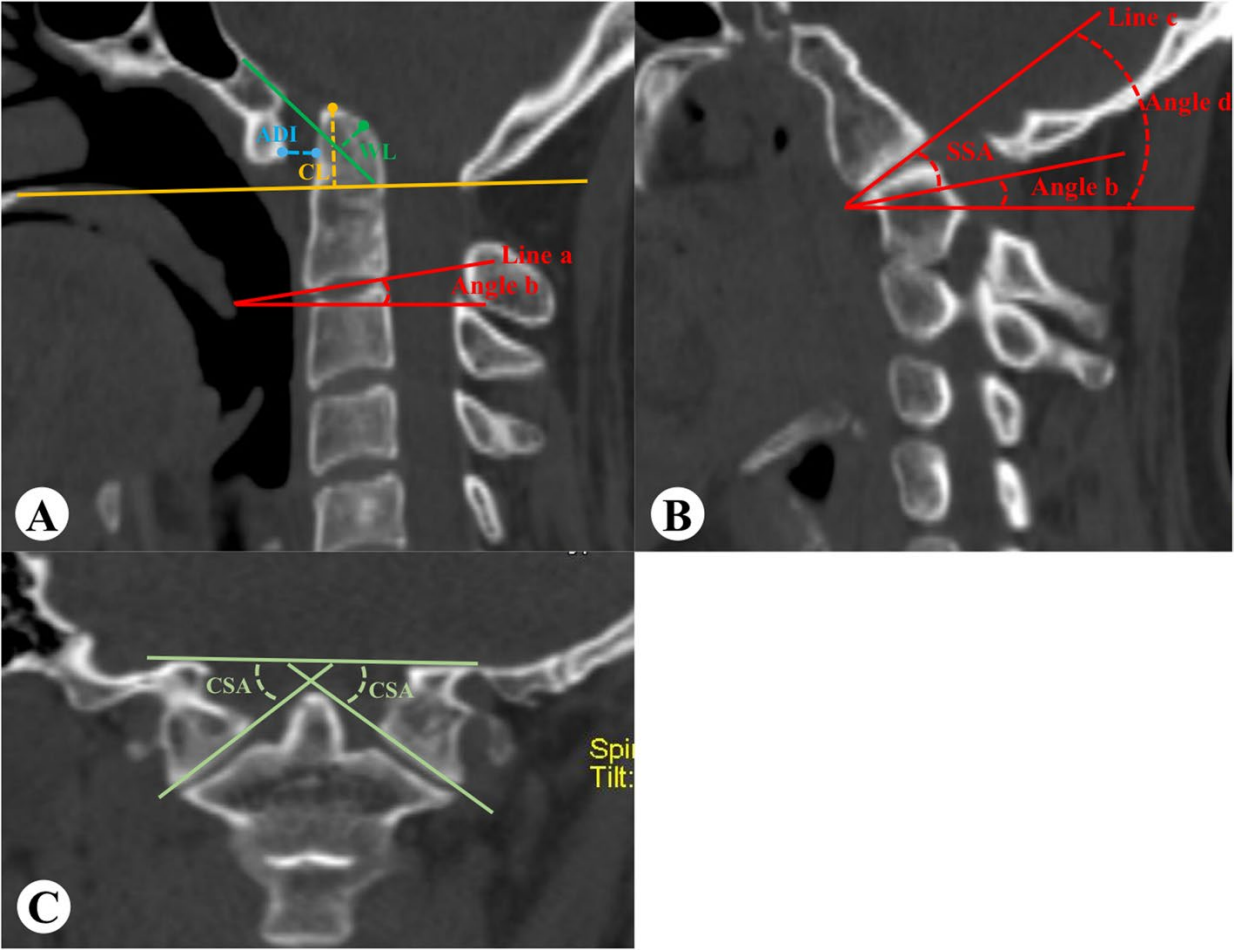
Measurement method. **A** On the midsagittal plane, distance-CL: draw a line from the posterior edge of the hard palate to the posterior upper edge of the foramen magnum occipitalis and measure the distance of the odontoid process beyond this line; Distance-WL: make the extension line of the clivus and measure the distance of the odontoid process beyond this line. ADI: measure the distance between the posterior cortex of the atlas anterior arch and the anterior cortex of the dentate process; SSA: make C2 lower endplate extension line (line a, and if C2-3 is not segmented, then use C3 lower endplate extension line) in the median layer, then make a horizontal line and record the angle between line a and horizontal line as angle b. Make upper LAA surface extension line (line c) in the clearest plane of LAA (**B**) and measure the angle between line c and horizontal line to obtain angle d. SSA= (angle d-angel b). **C** CSA was measured on the clearest plane of bilateral facets: the angle between the line connecting the uppermost point of foramen magnum on the coronal plane and the line connecting the inner and outer points of C1 lower LAA.

The distance of the odontoid tip beyond Chamberlain line (distance-CL) and Wackenheim’s line (distance -WL), the atlantodens interval (ADI), sagittal slope angle (SSA), and coronal slope angle (CSA) of the bilateral facet joints were measured using three-dimensional CT reconstruction of occipitocervical junction (Fig. 2). SSA was assigned a positive value if it tilted to the front. Conversely, if it tilted towards the rear, it was assigned a negative value.

The difference in SSA (difference-SSA) between the left and right LAA was calculated as the absolute value of (left SSA–right SSA). The mean SSA (mean-SSA) of the left and right LAA was calculated as (left SSA + right SSA)/2. Minimum-SSA (or minimum-CSA) were the smallest values in the left and right SSA (or CSA). Maximum-SSA (or maximum-CSA) were the largest values in the left and right SSA (or CSA). RD was calculated according to the precious study [9]:

$$\:RD=(1-\frac{cut\:off\:value-postoperative\:value}{cut\:off\:value-preoperative\:value})\times\:100\%$$

The cut-off values of ADI and distance-CL were both 3 mm, and of distance-WL was 0 mm. When the postoperative value was less than or equal to the cut-off value, RD was set to 100%. The closer the RD is to 100%, the better the reduction is. Preoperative reducibility was evaluated through preoperative dynamic lateral X-rays. If the preoperative overextension X-ray shows an ADI less than 3 mm, it is recorded as reducible. The above outcomes were measured separately by two researchers using PACS v4.0 (GE Healthcare, Milwaukee, WI, USA) software, and the mean values were included in the statistical analysis. If there was a large difference, a third senior surgeon would take the measurement and decide the value.

### Propensity score matching

To reduce the impact of potential confounding factors, such as sex, age, and body mass index (BMI) on the analysis of sagittal parameters, we used 1:1 propensity score matching, and the caliper was set to 0.05. The matching effect was evaluated using the chi-squared test, Wilcoxon signed-rank test, and standardized mean differences (maximum allowed of 0.1). This method could help us understand the differences in the LAA morphology between healthy people and patients with BI and AAD by reducing the confounding factors.

### Surgical method

The same surgeon performed reduction and occipitocervical fusion. Tracheal intubation was performed under general anesthesia, and the patient was placed in a prone position. Spinal cord function was monitored using somatosensory-evoked potentials. The head was fixed using a Mayfield head holder. Reduction was performed using a previously reported distraction and reduction technique [4, 9]. After reduction, internal fixation was locked, and the swallow tail-shaped massive iliac bone combined with granular autologous cancellous bone and demineralized bone matrix were used for bone grafting. After placing the drainage tube, the incision was closed layer-by-layer.

### Statistical analysis

The results were analyzed using the SPSS software (version 19.0). The Kolmogorov-Smirnov test was used to determine normality of the data. A t-test was used for normally distributed continuous variables. The results of continuous variables were expressed as mean ± standard deviation, and the count variables were expressed as the number of cases. χ^2^ tests were used for categorical variables. Pearson’s correlation coefficient was used to analyze the correlation between various imaging parameters. Univariate and multivariate logistic regressions were used to analyze the relationship between each index and RD. The receiver operating characteristic (ROC) curve was used to determine whether factors could be used as predictors of RD after surgery. Differences were considered statistically significant at *P* < 0.05.

## Results

### Demographics

A total of 613 controls and 62 patients with BI and AAD were included, among which 351 were males, and 324 were females. They had an average age of 51.7 ± 10.7 years. Before matching, the patient group included 21 males (33.9%) and 41 females (66.1%), with an average age of 49.9 years (18–72 years). Before matching, the control group included 330 males (53.8%) and 283 females (46.2%), with an average age of 51.9 (21–75) years (Table 1). Statistically significant differences were observed in sex and age between the two groups before matching.

**Table 1:** Comparison of LAA inclination between patient group and control group before matching

	Before matching				After matching			
	Patient group	Control group	*P* value	Std. Mean Diff.	Patient group	Control group	*P* value	Std. Mean Diff.
Sample size	62	613	-		62	62	-	
Sex (male/female)	21/41	330/283	0.003	0.293	21/41	24/38	0.5753	0.099
Age (year)			0.024	0.201			0.5086	0.009
< 45	25 (40.3%)	167 (27.2%)			25 (40.3%)	21 (33.9%)		
45–55	15 (24.2%)	254 (41.4%)			15 (24.2%)	22 (35.5%)		
55–65	16 (25.8%)	115 (18.8%)			16 (25.8%)	12 (19.4%)		
> 65	6 (9.7%)	77 (12.6%)			6 (9.7%)	7 (11.3%)		
BMI(kg/m^2^)			0.100	0.224			0.7043	0.068
≥ 23.9	20 (32.3%)	264 (43.1%)			20 (32.3%)	22 (35.5%)		
< 23.9	42 (57.7%)	349 (56.9%)			42 (57.7%)	40 (64.5%)		
Left-SSA (˚)	-18.4 ± 25.0	16.0 ± 6.2	< 0.001*		-18.4 ± 25.0	16.6 ± 6.4	< 0.001*	
Right-SSA (˚)	-20.7 ± 18.02	14.7 ± 6.4	< 0.001*		-20.7 ± 18.02	14.7 ± 6.7	< 0.001*	
Mean-SSA (˚)	-19.6 ± 18.9	15.3 ± 5.6	< 0.001*		-19.6 ± 18.9	15.6 ± 5.8	< 0.001*	
Difference-SSA (˚)	14.2 ± 16.4	4.8 ± 3.7	< 0.001*		14.2 ± 16.4	5.2 ± 3.8	< 0.001*	
Left-CSA (˚)	28.7 ± 16.8	23.2 ± 4.3	0.003*		28.7 ± 16.8	23.1 ± 4.1	0.011*	
Right-CSA (˚)	29.1 ± 14.8	23.7 ± 4.3	0.001*		29.1 ± 14.8	23.1 ± 4.2	0.003*	
Mean-CSA (˚)	28.9 ± 12.1	23.5 ± 3.2	< 0.001*		28.9 ± 12.1	23.1 ± 3.3	< 0.001*	
Difference-CSA (˚)	13.9 ± 14.9	4.3 ± 3.6	< 0.001*		13.9 ± 14.9	4.1 ± 3.1	< 0.001*	

*BMI* Body Mass Index, *SSA* Sagittal Slope Angle, *CSA* Coronal Slope Angle, *Mean Diff.* Standardized Mean Differences

*Indicates statistically significant differences (*p*<0.05)

### LAA inclination between patients and controls group

After propensity score matching, the 62 individuals in the control group were successfully matched to the 62 patients. After matching, there was no statistical difference in sex, age, or BMI between the two groups, and the standardized mean differences decreased to below 0.1, which indicated an acceptable matching effect. Left SSA and right SSA were significantly smaller in the patient group, while difference-SSA, left CSA, right CSA, and difference-CSA were significantly larger in the patient group (Table 1). Difference-SSA was more than 10 º in 28 participants in the patient group (45.2%). However, it was greater than 10 º in only six participants (9.7%) in the control group. Difference-SSA was significantly greater in the patient group than in the control group (14.2 ± 16.4 vs. 5.2 ± 3.8, *P* < 0.001). In the coronal plane, 28 patients (45.2%) had difference-CSA greater than 10 °, whereas only two participants (3.1%) in the control group had it greater than 10 °. The difference-CSA was significantly greater in the patient group than in the control group (13.9 ± 14.9 vs. 4.1 ± 3.1, *P* < 0.001).

### Relationship between LAA inclination and dislocation severity

Correlation analysis showed significant correlations between left-SSA, right-SSA, mean-SSA, minimum-SSA and maximum-SSA, and dislocation severity. The smaller the SSA value, the more severe the dislocation. However, no correlation was found between CSA and dislocation severity (Table 2). In addition, due to the anatomical complexity of BI with AAD, we further divided the patients into two groups based on the presence or absence of C1 occipitalization, and found that patients with C1 occipitalization had higher distance-CL (Supplement Table 1).

**Table 2:** Correlation between the LAA slope and the dislocation severity in patients with BI and AAD.

	Distance-CL	Distance -WL	ADI
Left-SSA (˚)	*r=*-0.340, *P =* 0.007	*r=*-0.251, *P =* 0.049	*r=*-0.269, *P =* 0.034
Right-SSA (˚)	*r=*-0.215, *P =* 0.093	*r=*-0.281, *P =* 0.027	*r=*-0.415, *P <* 0.001
Mean-SSA (˚)	*r=*-0.327, *P =* 0.010	*r=*-0.299, *P =* 0.018	*r=*-0.375, *P =* 0.003
Minimum -SSA	*r=*-0.273, *P =* 0.032	*r=*-0.293, *P =* 0.021	*r=*-0.302, *P =* 0.017
Maximum-SSA	*r=*-0.343, *P =* 0.006	*r=*-0.258, *P =* 0.043	*r=*-0.410, *P <* 0.001
Left-CSA	*r =* 0.120, *P =* 0.352	*r =* 0.232, *P =* 0.057	*r =* 0.216, *P =* 0.092
Right-CSA	*r=*-0.195, *P =* 0.129	*r =* 0.097, *P =* 0.453	*r =* 0.035, *P =* 0.787
Mean-CSA	*r=*-0.036, *P =* 0.783	*r =* 0.243, *P =* 0.053	*r =* 0.171, *P =* 0.184
Minimum-CSA	*r=*-0.129, *P =* 0.318	*r =* 0.212, *P =* 0.099	*r =* 0.057, *P =* 0.658
Maximum-CSA	*r =* 0.050, *P =* 0.698	*r =* 0.205, *P =* 0.110	*r =* 0.220, *P =* 0.086

Distance-CL, the distance of the odontoid tip beyond the Chamberlain line; Distance -WL, the distance of the odontoid tip beyond the Wackenheim’s line; *ADI* Atlantodens interval, *SSA* Sagittal Slope Angle, *CSA* Coronal Slope Angle

### Effect of LAA slope on the reduction degree and clinical outcomes after surgery

ADI, distance-WL, and distance-CL improved in all patients. Satisfactory horizontal reduction (RD ≥ 90%) was achieved in 52 patients (83.9%). At least 50 patients (80.6%) achieved a satisfactory vertical reduction (Table 3). Univariate logistic regression showed that smaller SSA and larger CSA were associated with worse horizontal RD (Table 4). However, no relationship was found between SSA and CSA and vertical RD. Because of the covariance among the various values of SSA (left SSA, right SSA, mean-SSA, maximum-SSA, minimum-SSA), we selected only the one with the highest correlation with RD for inclusion in the multivariate logistic regression analysis (same as CSA). Hence, the regression model would be unstable if there was collinearity among the multiple factors included in the logistic regression. Finally, we included minimum-SSA and mean-CSA in the multivariate logistic regression analysis (Table 5) and found that minimum-SSA was an independent predictor of horizontal RD; that is, the smaller the minimum-SSA, the worse the horizontal RD.

The overall incidence of complications was 11.3%, with a total of 6 patients experiencing dysphagia within 3 days after surgery, 1 patient developing pneumonia, and no patients experiencing neurovascular infections, wound infections, or device related adverse events. A total of 60 patients (96.8%) showed improvement in neurological function after surgery. The average postoperative JOA recovery rate was 69.4%. Because univariate regression found minimum-SSA and mean-CSA were related with the horizontal reduction degree, we conducted a targeted analysis of their relationship with the overall incidence of complications and JOA recovery rate. No significant difference was found in the overall incidence of complications compared to minimum-SSA (*p* = 0.9067) or mean-CSA (*p* = 0.5551). The average JOA recovery rate was not significantly related with minimum-SSA (*p* = 0.0642) or mean-CSA (*p* = 0.6793).

**Table 3:** Reduction degree after surgery

	Horizontal	Vertical	
	ADI	Distance -WL	Distance-CL
**RD ≥ 90%**	52 (83.9%)	50 (80.6%)	51 (82.3%)
**RD < 90%**	10 (16.1%)	12(19.4%)	11 (17.7%)

*RD* reduction degree, *Distance-CL* the distance of the odontoid tip beyond the Chamberlain line, *Distance -WL* the distance of the odontoid tip beyond the Wackenheim’s line, *ADI* Atlantodens interval

**Table 4:** Relationship between each index and RD ≥ 90% (univariate regression)

	Horizontal (RD of ADI)				Vertical (RD of Distance-CL)				Vertical (RD of Distance -WL)			
	β	**OR**	**95%CI**	*P*	β	**OR**	**95%CI**	*P*	β	**OR**	**95%CI**	*P*
**Left-SSA**	0.064	1.066	1.011–1.123	0.017	0.011	1.011	0.988–1.034	0.341	-0.006	0.994	0.963–1.026	0.691
**Right-SSA**	0.052	1.053	1.009-1.100	0.019	-0.006	0.994	0.957–1.033	0.764	-0.030	0.971	0.925–1.018	0.223
**Mean-SSA**	0.099	1.104	1.023–1.191	0.011	0.008	1.008	0.977–1.040	0.616	-0.022	0.978	0.930–1.030	0.400
**Minimum-SSA**	0.107	1.113	1.032–1.201	0.005	0.007	1.007	0.982–1.033	0.591	-0.004	0.996	0.964–1.028	0.789
**Maximum-SSA**	0.042	1.043	0.999 − 0.109	0.058	0.007	1.007	0.972–1.043	0.712	-0.039	0.961	0.914–1.011	0.128
**Left-CSA**	-0.085	0.919	0.860–0.982	0.012	-0.011	0.989	0.964–1.025	0.543	0.025	1.025	0.976–1.077	0.322
**Right-CSA**	-0.025	0.975	0.933–1.020	0.274	0.012	1.012	0.966–1.060	0.615	0.035	1.035	0.982–1.091	0.195
**Mean-CSA**	-0.081	0.922	0.867–0.981	0.010	-0.003	0.997	0.945–1.052	0.907	0.055	1.056	0.982–1.137	0.140
**Minimum-CSA**	-0.073	0.929	0.876–0.986	0.016	-0.003	0.998	0.948–1.050	0.924	0.041	1.041	0.981–1.106	0.184
**Maximum-CSA**	-0.050	0.951	0.907–0.997	0.036	-0.002	0.998	0.957–1.040	0.917	0.037	1.038	0.978–1.102	0.221
**Preoperative reducibility**	0.041	1.042	0.919 − 0.121	0.067	1.255	3.508	0.915–13.544	0.067	-0.575	0.563	0.107–2.951	0.496

*Distance-CL* the distance of the odontoid tip beyond the Chamberlain line, *Distance -WL* the distance of the odontoid tip beyond the Wackenheim’s line, *ADI* Atlantodens interval, *RD* Reduction degree, *SSA* Sagittal slope angle, *CSA* Coronal slope angle, *OR* odds ratio, *CI* confidence interval

**Table 5:** Relationship between each index and RD ≥ 90% (multivariate regression)

	β	OR	95%CI	*P*
**Minimum-SSA**	0.101	1.107	1.026–1.194	0.009
**Mean-CSA**	-0.028	0.973	0.891–1.062	0.536

*SSA* sagittal slope angle, *CSA* coronal slope angle, *OR* odds ratio, *CI* confidence interval

### Minimum-SSA and postoperative horizontal RD

**Fig. 3:**
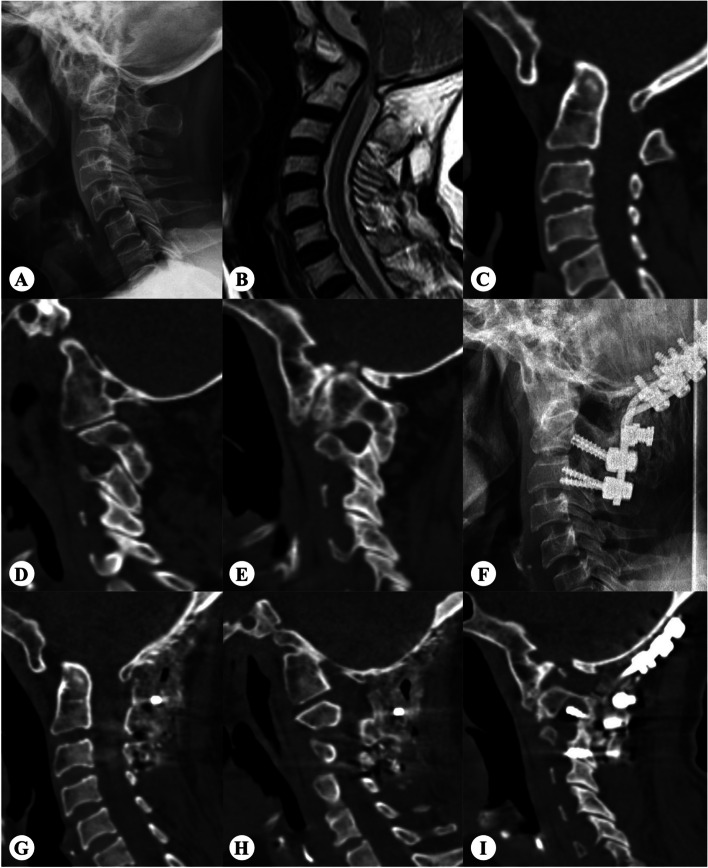
A 65-year-old female complained of numbness of limbs, unsteady walking, and neck pain. **A-C** Preoperative X-ray, MRI and CT three-dimensional reconstruction showed BI with AAD. **D** Preoperative CT showed right SSA was − 19.95˚ and (**E**) left SSA was − 66.52 ˚. Thus, the minimum-SSA was − 66.52 ˚. **F-G** Postoperative X-ray and CT three-dimensional reconstruction showed horizontal RD was not satisfactory. AAD was 6.25 mm, and RD was 51%. **H** The right LAA did not obstruct the horizontal reduction. **I** However, the left SSA was too small, which obstructed the horizontal reduction

We performed an ROC curve analysis to further explore the effect of minimum-SSA in predicting horizontal RD. The ROC curve for minimum-SSA as a predictor of satisfactory RD (RD ≥ 90%) yielded an area under the curve of 0.844 (95% confidence interval, 0.655–0.999; *P* = 0.001). A cut-off value of -40.2 was associated with 98.1% sensitivity and 80% specificity for predicting satisfactory RD. A typical case is shown in Fig. 3.

## Discussion

This is the first study investigating the effect of LAA morphology on the effect of surgical reduction. We found SSA is one of the independent factors related with severity of dislocation but not CSA. In addition, minimum-SSA was one of the independent predictors of satisfactory reduction.

SSA of a patient is smaller than that of healthy people and often tilts forward. The craniocervical region anatomy is complex. During human evolution, stability of the occipito-atlantoaxial junction was traded off to provide sufficient mobility to the head and neck. Additionally, the atlantoaxial spine supports the weight of the entire head [11, 12]. The mean-SSA of the control group in our study was 15.6 º, indicating that most healthy individuals have a posteriorly inclined LAA. This morphology prevents the occipital atlantoaxial complex from sliding forward and downward, thereby maintaining the normal anatomical state of the craniocervical junction area.

In contrast, mean-SSA in the patient group was − 19.6 º, indicating that their LAA was anteverted. In these patients, the occipital atlantoaxial complex tended to slide forward and downward at birth. This slippage gradually led to atlantoaxial dislocation during growth and development, with the odontoid process moving upward into the foramen magnum to compress the medulla oblongata and backward to compress the spinal cord. This also explains why such patients do not have symptoms at birth, but usually have corresponding symptoms in adulthood. Yin et al. [6] divided the LAA morphology into four types. Type I-III probably showed a progressive increase in anteversion, and type IV was defined as angular retroversion. Their study found that all patients with BI and AAD belonged to types I-III, and no atlantoaxial dislocation occurred in type IV. Chandra et al. [7] also found that the LAA of patients with BI was anteverted, although their measurement methods differed from those in this study. In addition, bilateral LAA of patients with BI and AAD were more asymmetric than that of healthy individuals. Currently, there is no definition for LAA asymmetry. In our study, 34 cases had a difference-SSA greater than 10 º, and 82.4% of them were patients with BI and AAD. There were 30 cases with difference-CSA greater than 10 º, and 93.3% of them were patients. This further indicated that the patient’s LAA was more asymmetric. LAA asymmetry may lead to torticollis, rotation deformity, and neck pain, which are consistent with the clinical manifestations of patients.

SSA was correlated with the severity of BI and AAD. The smaller the SSA, the more severe the LAA anteversion, which makes it easier for the atlas to slide forward and downward relative to the odontoid process, thereby causing the odontoid process to compress the spinal cord. Chandra et al. [7] found that the sagittal and coronal angles of the LAA were related to dislocation severity. However, interestingly, in our study, this phenomenon was only observed in the sagittal plane but not in the coronal plane. This is probably due to the opposite orientation of the right and left CSA in the coronal plane and the fact that the atlantoaxial spine is a bony, round-like structure with an almost constant diameter. Unless this ring elastically deformed in the coronal plane, it is difficult to cause relative sliding between the atlantoaxial axes. Therefore, there was no strong relationship between CSA and the severity of BI with AAD. It should be noted that SSA may not be the only factor affecting the severity of dislocation. We found that patients with C1 occipitalization have more severe vertical dislocation. But this is not the main purpose of this study, which requires further in-depth research in the future.

Minimum-SSA was a predictor of horizontal RD after surgery. For the first time, we found, through multivariate logistic regression and ROC curves, that the smaller the minimum-SSA, the worse the horizontal RD. During horizontal reduction, the LAA of the atlas moves backward relative to the LAA of the axis, and the excessive forward inclination of the LAA prevents this relative displacement. There is a significant obstructive effect whenever there is severe anteversion on one side of the LAA. Therefore, surgeons should carefully review patients’ atlantoaxial LAA morphology preoperatively. If the patient has a very small minimum-SSA, especially less than − 40.2 º, appropriate interventions may be needed, such as remodeling the LAA according to specific conditions. Many researchers have recently explored and developed various surgical methods for BI with AAD [8–18]. However, currently, there are no uniform surgical methods. Most surgeons prefer posterior direct surgical reduction and fixation for patients without bony fusion between the atlantoaxial planes. The reduction process often includes horizontal and vertical reduction. Therefore, our results could be applied to most reduction methods; nevertheless, further research from other surgical centers is required. We expect other surgical centers to report whether the minimum-SSA blocking effect also exists in other types of surgical reduction procedures. In addition, the impact of preoperative reproducibility on RD cannot be ignored, although we obtained a boundary *P*-value (0.067). Although minimum-SSA affects the reduction degree, there is not enough evidence to show that minimum-SSA affects postoperative JOA scores. Because some patients who have only achieved partial reduction may still have varying degrees of neurological function recovery.

### Limitations

This study has some limitations. First, because BI with AAD is not a common disease, it was difficult to collect a large sample size. Second, this was a retrospective study, and there may have been some unavoidable bias. Prospective studies are needed in the future. Third, there are various reduction methods for BI with AAD, and we expect other surgical centers to report the impact of SSA on horizontal RD. Finally, due to the fact that none of the patients in this study underwent joint release during surgery, it is difficult to evaluate the impact of joint release on RD.

## Conclusions

The SSA of patients with BI and AAD was significantly smaller than that of healthy individuals, showing an anteverted pattern. The LAA of patients was more asymmetrical than that of healthy individuals. Additionally, dislocation severity was associated with SSA, not with CSA. The smaller the SSA, the more the dislocation severity. Finally, minimum-SSA can be used as a predictor of satisfactory horizontal reduction. When minimum-SSA is less than − 40.2˚, horizontal reduction may be difficult, and the surgeon needs to consider alternative strategies.

### Supplementary Information

Supplementary Material 1.

## Data Availability

The datasets used and/or analysed during the current study available from the corresponding author on reasonable request.
