# Supplementary material for: Sagittal slope angle of lateral atlantoaxial articulation is associated with the severity of basilar invagination with atlantoaxial dislocation and predicts reduction degree after surgery
Source: BMC Musculoskelet Disord. 2024 Jul 24;25:577. doi: 10.1186/s12891-024-07696-4 (PMC11267687; doi:10.1186/s12891-024-07696-4)
Supplement: Supplementary file 1 — Supplementary Material 1. [file 12891_2024_7696_MOESM1_ESM.docx]

|  | C1 occipitalization | Not C1 occipitalization | *P value* |
| --- | --- | --- | --- |
| N | 56 | 6 |  |
| Distance-CL | 9.7±3.2 | 5.1±3.2 | 0.001 |
| Distance -WL | 6.3±2.8 | 4.9±4.2 | 0.272 |
| ADI | 6.3±2.1 | 6.0±2.5 | 0.748 |

Supplement table 1. Comparison between C1 occipitalization and severity of dislocation
